# Shelter Dog Census Changes Track Intakes but Resolve Slowly via Outcomes and Especially Adoptions

**DOI:** 10.3390/ani16152400

**Published:** 2026-08-04

**Authors:** Michael Loizos Mavrovouniotis, Kristen Hassen

**Affiliations:** 1 Social Compassion and SCIL, Laguna Beach, CA 92652, USA; 2 Cummings School of Veterinary Medicine, Tufts University, Medford, MA 02155, USA; kristen@outcomesforpets.com

**Keywords:** animal sheltering, in-care shelter population, animal shelter census, capacity for care, animal adoption, length of stay, live release rate

## Abstract

In animal shelters, the census (in-care population) changes when monthly intakes and outcomes are not balanced, and census changes may in turn affect subsequent intakes and outcomes. This observational, retrospective study examined these relationships using monthly dog statistics from the Shelter Animals Count database for United States government-related (GRO) and non-government (NGO) sheltering organizations over 2022–2024. Two linear regression models were applied: a concurrent model that decomposes single-month imbalances by intake and outcome type, and a cumulative model that estimates the effect of past census changes on subsequent monthly intakes and outcomes. Concurrent imbalances involved larger contributions from intakes than outcomes for both organization types. Accumulated past census increments dissipated predominantly through increased outcomes rather than reduced intakes, with adoptions making the largest absolute contribution; noncommunity live outcomes and nonlive outcomes were markedly overrepresented relative to their average shares. The implied time for 95% census recovery averaged approximately 7.6 months for GRO and 5.2 months for NGO. The marginal live release rate fell below the bulk average for GRO (85.8% vs. 90.1%) but not for NGO (96.8% vs. 96.6%). These findings argue against assumptions of fixed adoption or transfer capacity and support a view in which census perturbations are dampened (through shelter efforts) rather than permanent or unbounded.

## Simple Summary

Animal shelters take in dogs and later find outcomes for them, such as adoption or returning them to an owner. When more dogs arrive in a month than leave, the number of dogs in care—the census—goes up. We asked two questions: which arrivals and departures explain the month-to-month swings in the number of dogs in care, and how does a shelter work off a build-up of dogs once it has occurred? Using three years of monthly reports from shelters across the United States, we found that month-to-month swings track arrivals more closely than departures. A build-up of dogs, however, is worked off mainly by moving dogs out, especially through adoptions, over a period of several months. Shelters therefore appear to increase their efforts to secure live outcomes when they are fuller, and the number of adoptions a shelter achieves is not a fixed quantity. Understanding these typical patterns may help shelters respond to changes earlier and more effectively.

## 1. Introduction

Animal shelter intakes and outcomes by type do not remain constant from month to month. When, over a particular period, intakes exceed outcomes or outcomes exceed intakes, the animal census (in-care population count) changes. Thus, periodic imbalances cause the census to vary over time.

Changes in the census may, in turn, impact outcomes by type. A higher census may induce more adopters to visit (simply because they see more animals); induce shelters to expand marketing to adopters; induce rescue organizations to transfer animals out of the shelter; and result in increasing euthanasia as a last resort (with shelters interpreting their euthanasia criteria more broadly). Similarly, a reduction in census may reduce the number of adoptions and transfers and may reduce the number of animals euthanized.

The census may also influence future intakes. For example, a high census may result in the shelter taking measures to reduce intakes, including asking the public to hold found animals and try to find their owners, postponing or suspending intake of owner-relinquished animals, and other activities that fall under the broad label of managed intake [1,2]. Here, too, there are shelter decisions as well as external actors that may respond to census changes.

There are, then, bidirectional effects between census changes (intake–outcome imbalances) and intake and outcome counts by type. The effects vary by shelter, just like the average proportions among intake types or outcome types do. But, allowing for cross-sectional variations, the overall averages are useful for understanding the interactions between intakes, outcomes, and censuses in animal shelters. To the best of our knowledge, there is no published study modeling these dynamic effects in animal sheltering.

### 1.1. Direct and Indirect Influences

Adoption rates are particularly important. Along with the outcome type ‘return to owner’, or RTO, which is used for lost pets reclaimed by their owners, they constitute community live outcomes. These are animals that are permanently rehomed with an adopter or returned home to their original owner. The purpose of the mandatory hold period, which can range from three to 10 days or longer (depending on factors including state laws, local policies, and the animal’s microchipped status), is to give the owner time to reclaim their lost pet. After this period, the primary reason shelters hold animals is to give them a chance at a live outcome via an adoption or transfer.

Censuses and adoption counts influence each other, i.e., interact in both directions. There is a mechanical and precise effect of adoption count on censuses: each animal that leaves the shelter reduces the census that day. Since adoptions happen alongside other intakes and outcomes, these other events change the simple mechanics over a period of several days or weeks. If, for example, there are rescue organizations standing by to initiate a transfer of a particular animal if it is not adopted quickly, then the statistical effect of adoption on the census for the ensuing period is diluted. In other words, over time, the effect of any one adoption may be diluted to a smaller value because of the probability that some other outcome type would have occurred regardless.

In the other direction, the effects of census on adoptions are all indirect and may be positive or negative. A higher census may induce more marketing efforts and increase adopter traffic. If higher census involves more variety in available animals, the chances of an adopter finding a good match may increase. On the other hand, without a concomitant increase in resources, a higher census may also result in overcrowding, illness, and kennel stress [3], which translates to overall lower adoption appeal. Thus, in the absence of precise mechanical effects, there is a superposition of multiple indirect effects (mathematically positive or negative). Similar effects may occur for transfers to other organizations; i.e., the census may in some ways promote and incentivize and in other ways inhibit successful transfers.

### 1.2. This Study

This is an observational, retrospective study using dog statistics for sheltering organizations in the United States in the period 2022–2024 (i.e., after the core of the COVID-19 pandemic).

The data are obtained as monthly statistical aggregates from the Shelter Animals Count (SAC) database [4]. A previous study of shelter dynamics used SAC data to determine what portion of a change in the annual live release rate (live outcomes as a percentage of total outcomes) is attributable to each intake and outcome type [5], effectively decomposing live release rate changes into changes by intake and outcome type. (This decomposition approach focuses on associations among shelter variables. It differs from earlier research [6,7] examining temporal trends of intakes, outcomes, and live release rates.)

Here, we have a different objective and time scale. We study the relationship between census and each intake and outcome type in one-month intervals. We separate organizations into government-related organizations and non-governmental organizations, recognizing that the former are subject to additional legal obligations (such as intake of stray animals) and may be less susceptible to participation biases [5] (because of government transparency and public expectations). For each organization type, we determine the association of each intake and outcome type with concurrent (same-month) census changes, which shows what portion of census changes is explained by each intake and outcome type in monthly statistics. In the opposite direction of influence, we determine the magnitude of the impact of cumulative past census changes on each intake and outcome type. This then allows the comparison of intakes and outcomes (by type) at the margin of census changes to their respective bulk averages.

## 2. Materials and Methods

This study is limited to dogs in the United States. A study of cats would require additional modeling [5] to account for the seasonality of cat intakes and outcomes, shelter–neuter–return programs, or the nuances of traditional trap–neuter–return programs. (Unlike cats, dogs are not commonly returned to the field.) Our definition of government-related organizations (GROs) includes both government-operated and government-contracted shelters. We define non-government organizations (NGOs) as all other shelters and rescues. (Hereafter, we use the terms shelter and organization interchangeably, regardless of whether an entity operates dedicated facilities.)

### 2.1. Model

We begin by developing the model for how past census changes (the result of cumulative past imbalances) impact intakes and outcomes, which is the most challenging question in this study. Then, for consistency, we apply a similar model formulation to the question of how big a contribution intake and outcome types make to concurrent changes in the census. All intake and outcome types are mathematically handled the same way. We describe our methods using one outcome type, namely adoption, as the example.

Our formulation relates adoptions during a month to the census at the beginning of the month. While the census varies within a particular month and adoptions may be unevenly distributed within that month, the SAC data provides only monthly aggregates. Over a multi-month sequence, most of the variation in the census occurs across months rather than within a month.

We first form an equation for the variation in monthly adoptions over a sequence of monthly reports for a single shelter, then generalize across shelters.

#### 2.1.1. Use of Cumulative Imbalance for a Single Shelter

We examine consecutive months for each shelter to model the shelter around postulated steady-state census and monthly adoption counts. The steady-state values serve as a reference but do not need to be pre-computed. They are subsumed into other parameters, because they are not the primary goal of the regression. We are instead interested in the relationship between deviations (in census and monthly adoptions) from the reference.

We define these quantities, scaled by the shelter’s mean intakes and outcomes (as will be indicated in the Data subsection):

*A* = adoptions
*B* = imbalance = intakes − outcomes
*C* = census at the beginning of the period

Let the subscript *i* represent the index of the month in a sequence and the subscript *ref* the unknown reference levels. We postulate a linear relationship:

$$A_{i}-A_{ref}=K\left(C_{i}-C_{ref}\right)$$

If *K* = 0, then adoptions are not affected by the census.

If *K* > 0, a higher census results in more adoptions.

If *K* < 0, a higher census results in fewer adoptions.

The above equation does not mean that adoptions are proportional to the census. An equation of the form $A_{i}=KC_{i}$ is more restrictive and is not necessary for our model. We only assume that deviations from the reference are proportional. If *A* is a continuous function of *C*, this proportionality will hold at least for small deviations from steady state, but the magnitude and sign of *K* are unknown.

The census in one month can be related to the first month of the sequence:

$$C_{i}=C_{1}\;+\;\sum_{k=1}^{i-1}B_{k}$$

Substituting into the above adoption equation, we obtain

$$A_{i}=\left(A_{ref}+K{\;C}_{1}-K{\;C}_{ref}\right)+K\;\sum_{k=1}^{i-1}B_{k}$$

The first term, in parentheses, contains shelter-specific quantities, which cannot be disentangled using SAC data, and we simply aggregate them as a parameter *N*. (This parameter conveniently captures *A*_ref_, *C*_1_, and *C*_ref_ that were introduced only for the purpose of relating *A_i_* and *B_k_*). The second term uses the cumulative sum of imbalances up to and including the previous month, which we define as

$$M_{i}=\sum_{k=1}^{i-1}B_{k}$$

For regression purposes, then, we can simply write the adoption equation as

$$A_{i}=N+K\;M_{i}$$

#### 2.1.2. Regression Across Shelters

Since the reference levels depend on the shelter, *N* does as well. Since we are interested only in the average value of *K* (rather than shelter-specific behavior), we treat it as a single coefficient. Finally, in addition to an organization-dependent baseline, we include a period-dependent effect *P_i_*, with period *i* denoting the same month for all shelters. Thus, we model the adoptions *A_ji_* for shelter *j* and month *i* as a linear function of the corresponding cumulative imbalance *M_ji_* but with a shelter-specific intercept *N_j_* and period-specific effect *P_i_*, and a noise term denoted as ε*_ji_*:

$$A_{ji}=N_{j}+P_{i}+K\;M_{ji}+\varepsilon_{ji}\;(\mathrm{model}\;1:\;\mathrm{cumulative})$$

This is the core model used in the multiple linear regression. Regression software automatically adds an intercept term and uses one level in each categorical variable as a reference (which prevents collinearity between the sum of *N_j_* and the sum *P_i_*). A model of the same form applies to each intake and outcome type. We do not define new symbols for these or for the respective intercepts and slopes, as there will be separate rows for each intake and outcome type in the results.

We use this ordinary least squares formulation rather than a mixed-effects regression [8] for two reasons. Firstly, the assumption of normality of random effects does not hold here. Secondly, a mixed-effects regression would produce slopes *K* that are not linearly additive with respect to the aggregation of intake and outcome types. For example, if we chose to aggregate adoptions and returns to owners into a single outcome category (community live outcomes), the slope *K* would not equal the sum of the slopes of the two components. A mixed-effects regression was attempted, and these complications were observed; the results were nevertheless in qualitative agreement with those of the ordinary least squares regression presented here.

Since each shelter may be represented by an incomplete sequence, only some combinations of *j* and *i* have observations. Note that our motivation is the estimation of *K* and not the *N_j_* (and *P_i_*) covariates. Therefore, any offset of the *M_ji_* by a shelter-specific quantity, such as centering by each shelter’s mean, has no impact on the regression, as the covariate *N_j_* absorbs such shifts.

We refer to these regressions as cumulative, since they rely on the quantity *M_ji_*, which captures past census changes via cumulative monthly imbalances.

#### 2.1.3. Live Release Rate Metrics

In the cumulative regressions, the slopes capture the effects of census changes on intake and outcome types around the census reference level. These can be compared to the means given in the descriptive statistics for each intake and outcome type.

We define the reference live release rate (LRR) from the descriptive statistics as the ratio of live outcomes to all outcomes—agnostic to census and imbalance. We define marginal LRR as the corresponding ratio of the slopes.

The marginal LRR does not entail classifying individual animals as reference or marginal. It is a statistical measure of how a shelter’s whole animal population is affected by changes in census. This marginal LRR may vary by shelter, but our regression only computes the average behavior across shelters.

#### 2.1.4. Association of Intakes and Outcomes with Concurrent Imbalance

The instantaneous effect of each intake and outcome type on the census is straightforward. If nothing else changes, one extra intake or one less outcome causes an imbalance by 1, i.e., an increase in the census by 1. (We scale by a fixed quantity for each shelter, and 1 animal translates to a change of 1/scale in the scaled variable space, irrespective of intake or outcome type, and the effect remains the same.)

For a monthly interval, however, intake and outcome types are correlated. For example, within a given month, an extra stray intake has some probability of a fast outcome of a return to the owner, and its expected effect on the imbalance (census change) is less than 1. The one-month reporting interval matters. Events such as the return of stray animals to owners occur at time scales well below a month, and that creates a (potentially substantial) automatic deviation from the instantaneous mechanistic effect.

What is of interest is a descriptive variance/covariance decomposition of the imbalance into intake and outcome types that allows for the correlations observed in practice. To determine the contribution of each intake and outcome type to concurrent (same-month) imbalances, we follow the approach previously used for changes in LRR [5]. This consists of regressing each intake and outcome type as a dependent variable against the concurrent imbalance. For consistency, we use the linear form of model 1 and simply replace the cumulative past imbalance with the concurrent monthly imbalance. For adoptions, then,

$$A_{ji}=N_{j}+P_{i}+K\;B_{ji}+\varepsilon_{ji}\;(\mathrm{model}\;2:\;\mathrm{concurrent})$$

Each intake or outcome type is handled the same way, and adoptions only serve as an example for the mathematical formulation.

We refer to these regressions as *concurrent*, since they use the quantity *B_ji_*—the one-month imbalance (census change)—for the same period as *A_ji_*. Conceptually, model 2 captures a causal direction opposite to that of the cumulative model 1. Model 2 is intended to capture how adoptions contribute to census changes, while model 1 aims to show how census change impacts subsequent adoptions. Model 2 is a descriptive decomposition, while model 1 is a forward estimation.

We do not define new symbols for the intercept and slope, since one regression (for each of the two models) is done separately for each intake and outcome type. The generic symbols in the mathematical derivation are already stand-ins for several regressions.

With ordinary least squares, the sum of the *K* estimates for intakes minus outcomes is equal to 1, because the concurrent imbalance is defined as the sum of intakes minus the sum of outcomes. This identity would not hold if we used mixed-effects regression, and that is one reason for opting for ordinary regression.

### 2.2. Data

We obtained SAC monthly organization-level reports [4] on dog intakes and outcomes for GROs and NGOs in 2022–2024. If an organization changed status between GRO and NGO during this period, it was treated as two organizations. We used the intake and outcome fields defined by SAC but ignored the separation into youth and adult because this is only available for some shelters. We excluded owner-requested euthanasia because it is currently classified by SAC as a service distinct from intake and outcome data. Excluding reports with zero intakes or outcomes, there were 27,488 reports for GROs and 36,442 for NGOs.

In some monthly reports, large values (≥50) in the transferred_in_undesignated_total field were identical to another, geographically specific incoming transfer field, raising a concern over double-counting errors [5]. These and reports with similar anomalies in outgoing transfers were discarded out of an abundance of caution. This occurred in 47 distinct GROs and 52 NGOs, with one of each leaving the sample altogether. This left 27,376 monthly reports for GROs and 36,275 for NGOs.

Reports with missing or negative counts in any intake or outcome type were removed. In the case of multiple reports referring to the same organization and month, we retained the report entered in the database most recently. Reports with fewer than 10 intakes or 10 outcomes were removed. Organizations with fewer than 6 valid monthly reports were removed. This left 25,755 reports for GROs and 26,219 for NGOs, with the drop in NGOs caused by their smaller size and sparser reporting.

#### 2.2.1. Sequences of Monthly Reports

We grouped the reports for each organization and detected sequences of consecutive reports. For each organization, we retained only the longest sequence, requiring a minimum of 6 reports. (In case of a tie, we used the most recent sequence.) These sequences are not, in general, aligned or of equal length across organizations. The sequences contained 22,697 reports from 860 GRO and 19,969 reports from 878 NGO.

#### 2.2.2. Aggregating and Scaling

We collapsed SAC’s intake and outcome types as follows. For intakes, we retained stray and owner-relinquished types but grouped the rest into one type of remaining intake. For outcomes, we retained adoption and return to owner from SAC, grouped all types of death or loss of animal into nonlive outcomes, and grouped the rest of the (live) types into noncommunity live outcomes. This last category consists predominantly of transfers to other organizations; as a shorthand in the text, we may refer to these simply as (outgoing) transfers. The aggregation facilitates interpretation of the results. For each month, we defined the imbalance as total intakes minus total outcomes. Positive imbalance means an increase in the census, while negative imbalance means a decrease.

Since we seek relationships compatible across shelters of different sizes, we scaled all counts of intakes and outcomes by a scale shared between intakes and outcomes, namely the mean across monthly intakes and outcomes for the whole sequence. Each shelter has one sequence of monthly reports, and thus a single scale for all its intake and outcome counts.

The slight variation in the number of days in a month is less important than other effects we are neglecting, such as organization-specific holidays, hours of operation of intakes, and hours of visitation by day of the week. In the interest of simplicity, we treated a month as a time unit without adjusting intake or outcome numbers by the number of days in the month.

#### 2.2.3. Cumulative Imbalances

For a given month, we computed the cumulative imbalance as the sum of the imbalances of all prior months. This represents the total census change from the beginning of the sequence to the beginning of the month it refers to. It is zero for the first month in the sequence. It is scaled, as intakes and outcomes are, by the shelter’s mean monthly intakes and outcomes.

As the model involves linear impacts of cumulative census changes, we want to retain only moderate changes for each organization. To facilitate this, we mean-centered the cumulative imbalance for each shelter, recalling that our model and data do not involve absolute census levels. We then retained only the months for which the cumulative imbalance is in the range −0.4 to +0.4 and required that an organization have at least two months in this range. The bound is intended to exclude major changes in shelter facilities, contractual arrangements, service areas, or unusual events with extreme census changes (such as natural disasters). When an organization ends up with only one month of data, that month is noiselessly captured in *N_j_* without contributing to the coefficient *K* and is therefore of no use.

The regression data set has 16,257 reports from 831 GROs and 12,906 reports from 845 NGOs. Though we use ordinary linear regression estimates, we cluster standard errors [9] by organization to allow arbitrary within-organization correlation and heteroscedasticity. If the number of organizations (clusters) is *G*, the inference is based on t-distributions with *G* − 1 degrees of freedom. We therefore have 830 degrees of freedom for GROs and 844 for NGOs.

### 2.3. Descriptive Statistics

For consistency between concurrent and cumulative regressions, the same filtered sample described above is used for both. Its descriptive statistics are shown in Table 1. The maximum values exceed 1 because a month is not scaled by its own intakes or outcomes but rather by the average intakes and outcomes of a sequence of (consecutive) monthly reports of that shelter. If intakes and outcomes are high in one month and low in other months, then scaled values can be greater than one. Because we filtered by the cumulative imbalance at the beginning of the month (mean-centered, as indicated above) but not by that month’s own imbalance, large magnitudes can also occur in the single-month imbalance.

Intakes in GRO shelters are dominated by strays at 0.587. Intakes in NGOs are dominated by remaining intakes at 0.499 (principally incoming transfers). Adoption outcomes are dominant in NGOs (0.806) but less so in GROs (0.452). Returns to owners are much higher in GROs (0.274) than in NGOs (0.053).

The implied average LRR computed from these descriptive statistics is 90.1% for GROs and 96.6% for NGOs.

Because we used a single scale shared between intakes and outcomes for each shelter’s sequence of monthly reports, the mean imbalance is not zero. GROs have mean total intakes of 1.007 and outcomes of 0.992, while NGOs have 1.000 and 0.992, respectively.

### 2.4. Software and Statistical Analysis

Initial data processing used Python 3.11.13 (Spyder IDE 6.0.8; MacOS Tahoe 26.4.1) with the modules numpy and pandas (version 2.3.2).

Regressions and statistics used the R language [10] version 4.4.2 on RStudio 2025.09.1+401, and the library package estimatr (version 1.0.6) [11]. Using this package, we clustered standard errors by organization using the CR1 estimator with the Stata-style small-sample correction [9,12]. Inferences are thus based on t-distributions with *G*−1 degrees of freedom, where *G* is the number of organizations. Given the large number of clusters (831 and 845), more advanced small-sample refinements to the estimator [12] are immaterial, and CR1 is sufficient.

Tests are at the 0.05 significance level, and 95% confidence intervals (CI) shown are two-sided (2.5–97.5%). The objective of the study is to estimate average slopes of intakes and outcomes regressed on the census. The shelter and period parameters are introduced to remove other effects. For our purposes, it does not matter whether these remaining parameters can be determined reliably, because we are not making predictions by shelter or period. Thus, we report only t-statistic, *p*-value, and CI for one slope estimate in each regression (rather than overall regression tests, which would be influenced by *G* shelter parameters and 36 period parameters).

## 3. Results

The relationship between changes in census and changes in intake or outcome counts is modeled linearly and symmetrically. Results should be understood to apply equally to positive or negative imbalances (increases or decreases in the census). Stating that a census increase is associated with an increase in adoptions is, within our model, equivalent to stating that a census decrease is associated with a decrease in adoptions.

Recall that we use two types of regression models. The concurrent regressions (model 2) decompose the single-period imbalance (census change) into intake and outcome types to explain how imbalances arise. The cumulative regressions (model 1) capture the effect of past cumulative imbalances (census changes) on intakes and outcomes to shed light on how a change in census dissipates via intake and outcome changes. The regressions were done on the same filtered data set, whose descriptive statistics are shown in Table 1.

Reversing the order of presentation in the methods, we present concurrent regression results (model 2) before cumulative regressions (model 1) because the concurrent results are simpler to understand.

### 3.1. Concurrent Regression

**Figure 1:**
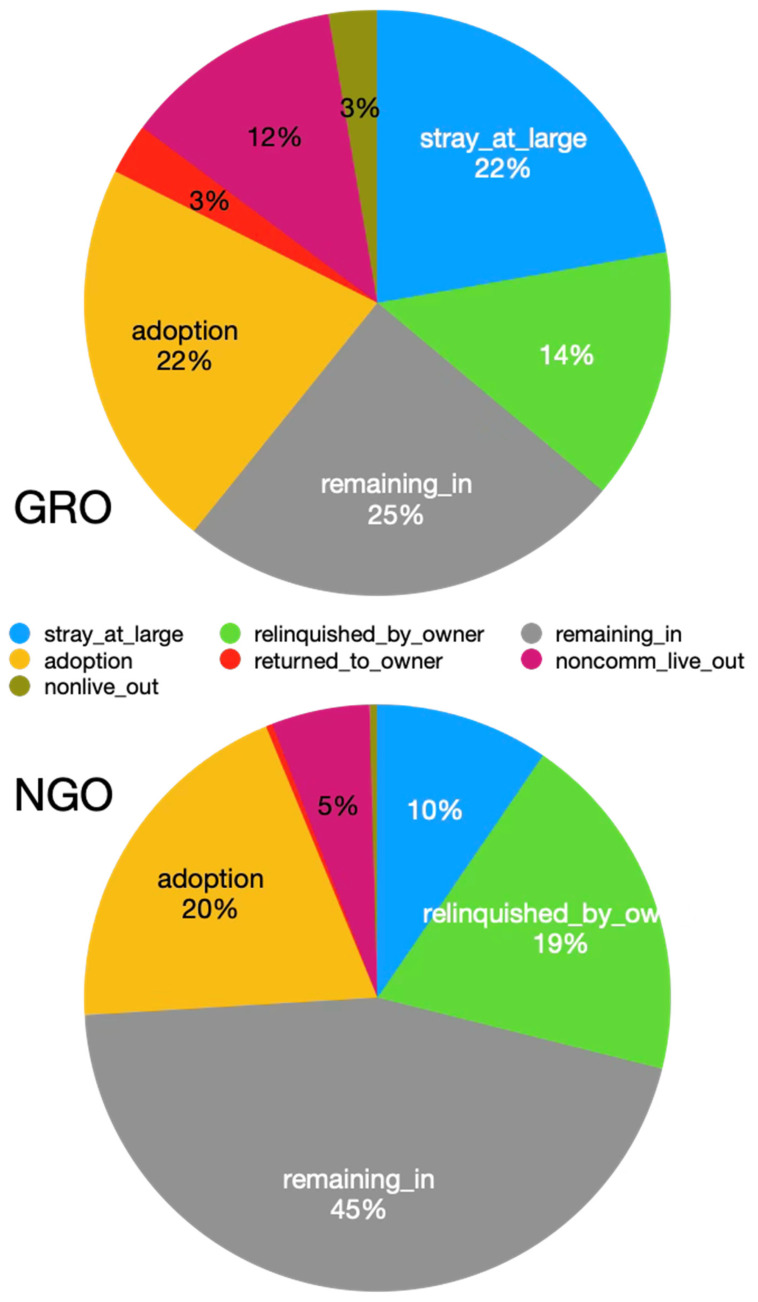
Contributions of intake and outcome types to concurrent imbalance (census change) as a percent of the total. A census increase consists of some combination of intake increases and outcome decreases. Intake types use white text inside the chart, and their underlying values are positive; outcome types use black text, and their underlying values are negative. Portions greater than 15% of the total are named inside the chart. Data are from Table 2.

As expected for a decomposition, the regression coefficient on concurrent imbalance is positive for intake types and negative for outcome types (Table 2); i.e., increases in intakes and decreases in outcomes correspond to increases in censuses. All slopes are statistically significant at the 5% level (and for most also at the 1% level). To facilitate comparisons, we also graphically present the relative magnitudes of contributions as fractions of the total (Figure 1). The percentages in Figure 1 track the values in Table 2, because the sum of the intake estimates minus the sum of the outcome estimates is equal to 1, based on the definition of the concurrent imbalance and our use of a single scale shared across intakes and outcomes.

For GROs, increases in remaining intakes (0.247), increases in strays (0.222), and decreases in adoptions (−0.216) each account for at least 0.2 of the concurrent imbalance. Increases in relinquished by owner (0.139) and decreases in noncommunity live outcomes (−0.121, which are principally outgoing transfers) each account for at least 0.1. In total, the split between intake and outcome types is 61% to 39%.

For NGOs, the dominant effect on imbalance is increases in remaining intakes (0.452), followed by adoption (−0.197), which is roughly tied with relinquishment by owner (0.194). The split between intakes and outcomes is 74% to 26%.

For both categories, imbalance is more strongly associated with intakes than outcomes.

### 3.2. Cumulative Regression

A positive past cumulative imbalance (i.e., increase in census) at the beginning of the month is associated with a decrease in each intake type and an increase in each outcome type (Table 3). However, the reductions in intake types are generally lower in magnitude and are statistically significant in only two out of six instances. No intake is statistically significant for GRO. Increases in outcome types are larger and are statistically significant in seven out of eight individual instances (all except returned to owner in GRO). Across the board, higher census is associated with elevated subsequent outcomes of each type and to a far lesser extent with reduced intakes.

**Figure 2:**
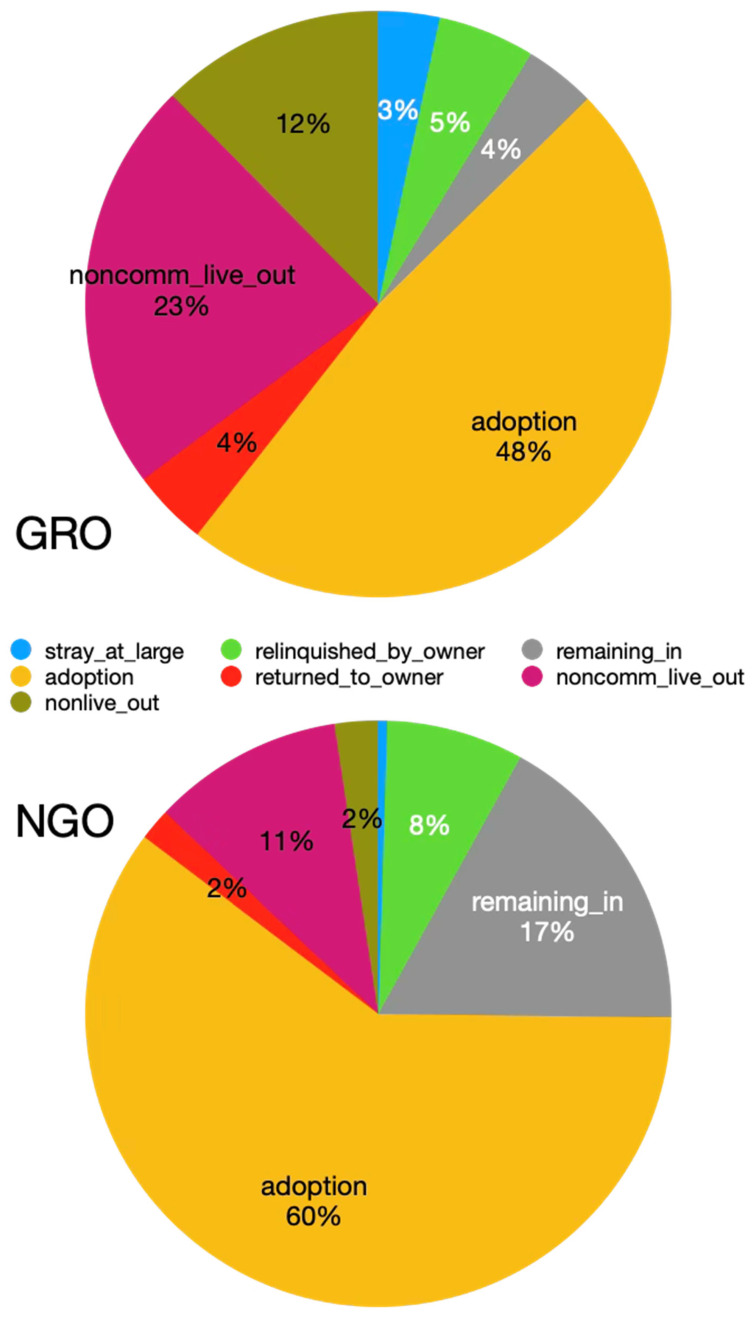
Fraction of the total impact of census change (past cumulative imbalance) by intake and outcome type. A census increase leads to decreases in intakes and increases in outcomes. Intake types use white text inside the chart, and their underlying values are negative; outcome types use black text, and their underlying values are positive. Portions greater than 15% are named inside the chart. Data from Table 3.

To facilitate comparisons, we graphically present the relative magnitudes of contributions as fractions of the total (Figure 2). The percentages in Figure 2 differ from the values in Table 3, because the summation of the effects of past imbalances is not equal to 1; i.e., the past census changes resolve only partially in each month. (The portion that resolves equals the −0.325 slope given for the month’s imbalance in Table 3.) The split between intakes and outcomes is 13% to 87% for GROs and 25% to 75% for NGOs.

Adoption dominates. For each additional dog in the census (as of the beginning of the month), there are 0.156 adoptions (48% of the total effect) for GROs and 0.264 (60%) adoptions for NGOs. For GROs, the next largest effect is noncommunity live outcomes (0.074; 23%), while for NGOs, it is remaining intakes (−0.075, 17%). This is consistent with NGOs having more discretion over intakes than GROs.

For GROs, the largest intake category is owner relinquishments (−0.017, 5%, but not statistically significant), suggesting that this is a weak lever for managing the census. Among outcome types, the smallest increase is dogs returned to owner (0.014, 4%). All intakes combined (0.041) approximately equal the nonlive outcome effect (0.040) and are dwarfed by adoptions (0.156).

#### 3.2.1. Marginal LRR

Nonlive outcomes play only a small part in dissipating census changes, at 0.040 for GROs and 0.011 for NGOs. The marginal LRR implied by these coefficients is 85.8% for GROs and 96.8% for NGOs. The marginal rate is lower than the bulk rate (90.1%, computed from the descriptive statistics) for GROs but virtually unchanged (from 96.6%) for NGOs. In effect, increases in census have a small negative impact on LRR in GROs but virtually no effect in NGOs.

#### 3.2.2. Relaxation Time Scales

The total intakes minus the outcomes equals the imbalance. Table 3 includes regression results for the imbalance. (The estimate itself can also be computed from the intake and outcome types, but a separate regression is a convenient way to produce a confidence interval and *p*-value for the imbalance). In one month, the accumulated imbalance is dissipated by −0.325 in GROs and by −0.439 in NGOs. Treating time as a continuous variable, these imply a timescale for census recovery from a perturbation: −1/ln(1 − 0.325) = 2.5 months for GROs and −1/ln(1 − 0.439) = 1.7 months for NGOs, where ln denotes the natural logarithm. These are exponential decay time constants, not the horizon for complete resolution. A resolution of 95% of a census change requires three times as long: 7.6 months for GROs and 5.2 months for NGOs.

#### 3.2.3. Relative Slopes

We can compare the slopes in Table 3 to the means in Table 1 to determine over- or under-representation of each intake and outcome type in the dissipation of the cumulative imbalance. An agnostic default would be that total intakes and total outcomes contribute equally and, within these, each type contributes in proportion to its existing average. For example, for adoptions in GRO, take the 0.325 overall dissipation on the imbalance row of Table 3, divide by 2 to attribute half of it to outcomes, then multiply by 0.452 (the adoption average from Table 1). This gives 0.0735 as the default contribution adoptions make to the overall 0.325 imbalance dissipation. Then the ratio of the observed coefficient of 0.156 to this default gives us 2.12, meaning that at the margin of census changes, adoptions contribute twice as much as would be proportionately expected.

**Figure 3:**
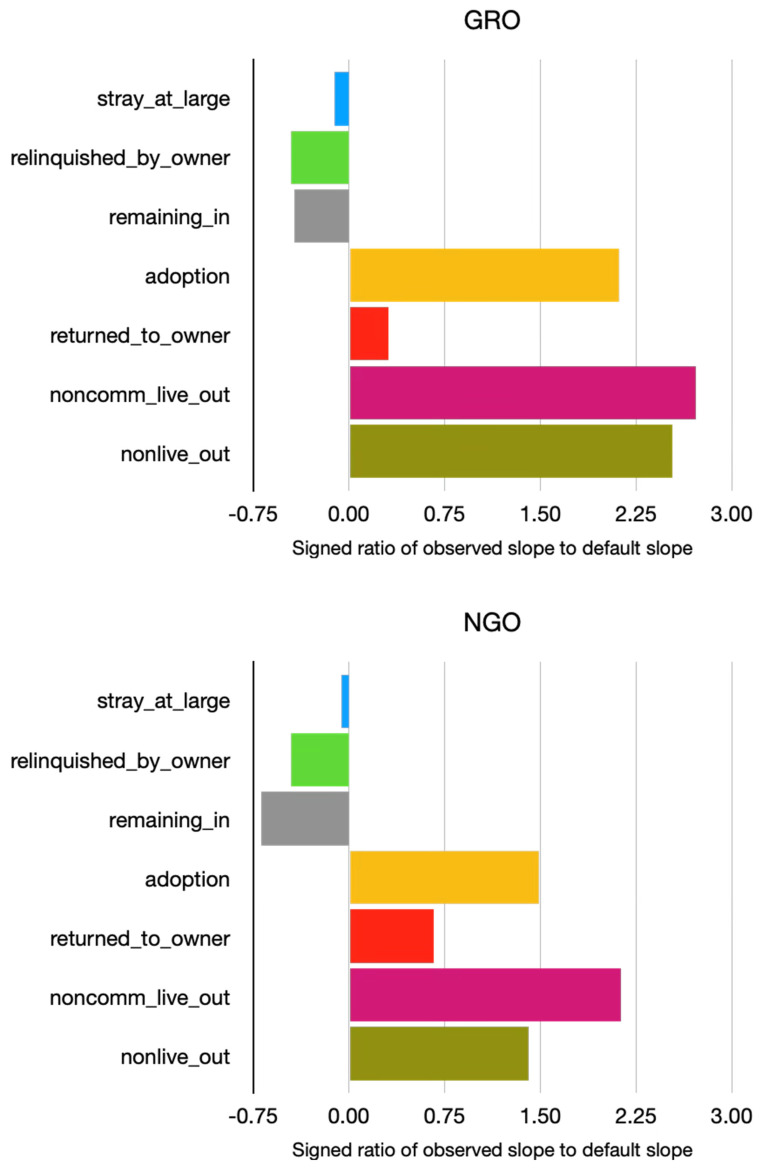
Over- or under-representation of each intake and outcome type in the dissipation of the cumulative imbalance. The ratio compares the impact of past census changes on intake and outcome types to an agnostic default. The default apportions the total impact equally between total intakes and total outcomes and, within each of these, to each type in proportion to its observed average value. The sign is preserved as that of the unscaled impacts and is negative for intakes and positive for outcomes. (A census increase leads to decreases in intakes and increases in outcomes.) Absolute magnitudes exceeding 1 signify overrepresentation in the impact of census changes. Magnitudes below 1 signify underrepresentation. Data are from Table 4.

Examining all intake and outcome types (Table 4, Figure 3) for GRO, we find that adoption, noncommunity live outcome, and nonlive outcome all contribute at more than twice the default rate, while returns to owners and all intake types contribute less than half of their default levels.

For NGO, noncommunity live outcomes contribute at twice the default level, adoptions and nonlive outcomes contribute at over 1.4 times their default, and other types are under-contributing.

The ratios in Table 4 and Figure 3 complement those in Table 3 and Figure 2, because they show changes relative to the bulk values. For example, for GROs, nonlive outcomes have the largest ratio in Table 4, meaning they experience the largest overrepresentation at the margin, relative to their normal level. But because their level is much lower than adoptions to start with, they remain a small portion of outcomes at the margin.

## 4. Discussion

For dogs in US-based animal shelters, this study suggests that the two directions of influence between census and the flow of animals behave differently. The first direction is how each intake and outcome type contributes to a change in census within a single month. The second is how a census that has already been built up or brought down over several months gets resolved.

Within a single month, the decomposition of the change in census involves larger components for intakes than for outcomes (Table 2, Figure 1), and adoption is the only outcome type that contributes substantially. Once a census change has accumulated over several months, shelters resolve it mainly by moving animals out. The accumulated census changes result only in small changes in intake. Adoptions make the largest absolute contribution. Transfers and nonlive outcomes are higher than their reference levels; i.e., they are elevated in response to a higher census and reduced in response to a lower census. Though nonlive outcomes are higher than what one would expect purely proportionally (Table 4, Figure 3), in absolute terms, a census increase nevertheless is associated with far more extra adoptions than extra nonlive outcomes (Table 3, Figure 2). Returns to owners stay flat across every analysis, which suggests that this outcome type is not responsible for census changes and that shelters have limited ability to move that number up or down in response to the census.

The differences between GROs and NGOs appear connected to the fact that NGO entities have control over what animals enter and when the intakes occur, and their outcomes are dominated by adoptions. The mandates or contracts under which GROs operate vary by jurisdiction. Even when GROs are permitted to divert or postpone intakes and attempt to do so, they must usually admit stray dogs. If GROs do not accept owner relinquishments, it is likely that at least a portion of these animals enter the system as strays when they are either abandoned or their owners claim they are unowned, rendering this mechanism less effective. NGOs have a higher level of incoming transfers (remaining intakes in Table 1 are half the NGOs’ total intakes), and their multi-factor decisions to accept them [13] can take census into account.

Our results indicate that census changes (such as those caused by a temporary spike in intakes) resolve at implied time scales of a few months, with somewhat elevated nonlive outcomes for GROs but live outcomes still dominating. In the cumulative regression, the slope of the imbalance (which is the sum of the slopes of intake types minus outcome types) captures how fast census changes resolve. The time scale implied by this slope is 2.5 months for GROs and 1.7 months for NGOs and would achieve resolution of 63% of a census perturbation. For 95% resolution, 7.6 months are required for GROs and 5.2 months for NGOs. The resolution is expressed as a percentage of the perturbation and would thus leave a larger residual for larger perturbations. If, theoretically, each animal had outcome rates that could vary over its stay but were independent of the census, then the time scale of resolution of census changes would roughly correspond to the average length of stay (LOS). Reported lengths of stay for GROs are usually below one month [14,15,16], but our observed time scale is much longer, implying that census changes do affect each animal’s outcome rates and that average LOS rises when the census increases.

This GRO time scale could correspond to the heavy (right) tail often noted in LOS distributions [15,17]. An increase in census may be accompanied by changes in the composition of the population in care. When insufficient adoptions lead to higher census, this may indicate that more dogs in slow-to-adopt categories (such as large dogs [16] or breeds overrepresented in shelters) have entered the census. That composition change would then lead to lower marginal LRR (for GROs) and longer stays [14,17,18], and the latter would lead to a longer time scale of resolution of the census change. This is only a hypothesis. It cannot be tested with the aggregate SAC data used in this study, but it is consistent with the observed time scales.

The fact that the marginal LRR is lower than the corresponding bulk average is not surprising. If resources and staff for animal care, adoption counseling, and marketing do not grow sufficiently to fully cope with census increases, then each animal will have somewhat worse prospects when the census is higher and better if the census is lower. This means that overall LRR is likely to decrease as census increases, causing lower marginal LRR—as well as longer LOS, as discussed above.

This effect does not automatically mean that the change in census is detrimental to animal welfare. The assessment of marginal LRR entails a counterfactual, namely how a shelter could prevent census changes. If, hypothetically, a shelter used euthanasia as the primary means of reducing the population, then the marginal LRR would not be merely lower than the reference LRR but very low in absolute magnitude. The modest drop for GROs suggests that shelters substantially accommodate census changes, but not without consequences. Though GROs handle the higher census largely through increasing live outcomes, a slightly higher percentage of the animals end up euthanized than otherwise would. For NGOs, marginal LRR matches the bulk average, which is consistent with planning intakes to match anticipated adoptions.

### 4.1. Relation to Shelter Constraints and Programs

There can be an upper bound on the census, based on a shelter’s resources [19,20]. If a GRO shelter is at capacity, it may temporarily limit intakes to those legally mandated (while NGO shelters reduce intakes at will as they are not mandated to take any animals). If, in that scenario, a GRO declines an owner relinquishment, it would seek to avoid euthanizing an animal already in care (if there is not enough space for both). To avoid this impasse, a shelter may take anticipatory management measures when the census reaches some level below the hard limit [2]. In our study, we have no information on where the shelter census stands with respect to hard limits or action points. These are governed not only by facility space and staff resources but also by policies. Some shelters have a tolerance for co-housing, and some do not. Some have access to as-needed foster care as an alternative to kennel housing. Our results shed no light on upper census bounds.

Our study shows that, in a statistical sense, adoption flow is not rigid. It therefore lends indirect support to programs that increase adoptability (enrichment and behavior programs) or facilitate the adoption process itself. If adoption numbers were rigid, there would be an internal competition among adoptable dogs (with advancement for one dog only at the expense of another). Our study suggests that dog adoptions and transfers (but not returns to owners) respond to census changes, and that is likely due to shelter needs, efforts, and policies. Since our study is observational, the results automatically incorporate shelter efforts to adapt to census changes, as well as the response of external actors such as adopters. One external channel requires no awareness of census changes at all: adopters respond to the population they encounter, and a higher census mechanically presents more candidates. (There can also be a countervailing effect of too many choices making adoption decisions more difficult.) Beyond this exposure effect, however, awareness of census changes is most immediate and unequivocal within the shelter itself, and it is reasonable to assume that shelter decisions, both proactive and subconscious, are a primary factor. It is likely that shelters increase active promotion of animals when the census rises. The study tells us not what would happen if shelters remained oblivious to census changes, but rather what happens on average, given common shelter efforts to respond to census increases. While we cannot quantify the extent of such efforts, our results imply that they are productive. This study’s retrospective quantification of the impact of census changes on intakes and outcomes may assist shelters in modifying their responses, activating them earlier, and (for GROs) obtaining authorization from local government for programs that impact a shelter’s finances (such as off-site events).

### 4.2. Dynamic and Stochastic Views

To clarify the consequences of the cumulative model, it is useful to discuss the fundamental reasons why the slopes of certain live outcome types should not be zero. The live outcomes of interest here are adoptions and other live outcomes, the latter being predominantly transfers. (We do not include animals returned to owners because this outcome type does not appear to respond to census changes.) Below, we contrast the findings in this study to the alternative of treating these two live outcome types as fixed shelter capacities unresponsive to censuses.

If a shelter is at a steady state and experiences no random fluctuations, the relationship between live outcome counts and censuses is not observable. With fixed (daily) intakes, outcomes, and censuses, the numbers of adoptions and transfers indifferently represent either fixed capacities or census fractions. But when we consider system dynamics and stochastic variation, fixed capacities for adoptions and transfers become problematic.

#### 4.2.1. Dynamic View

Suppose that, starting from an initial steady state, a shelter experiences a step increase in intakes; i.e., monthly intakes rise to a permanently higher level. Under an assumption of fixed capacity for live outcome types, either the census must monotonically increase without limit (not a new steady state), or the entire change in intakes must be diverted to euthanasia as the only outcome over which a shelter has direct control. In practice, shelters experience changes in intakes, and they reach another steady state without diverting the increase to nonlive outcomes. (A 5% increase in intakes does not, in practice, lead to a five-point loss in LRR, and a 5% decrease in intakes does not translate to a five-point gain in LRR).

Less dramatically, suppose there is a temporary spike in intakes, e.g., caused by an unusual weather event. Additional animals enter the shelter, but the daily intakes then return to the normal rate. If live outcome capacity is rigid, the additional animals must either experience other outcomes (which again raises the question of euthanasia) or result in a permanent increase in the census. The latter implausibly would mean that a shelter taking in 10 extra dogs this week will have a census elevated by 10 not just for a few months but for the indefinite future. The findings of this study suggest that temporary spikes resolve in a few months, predominantly through adoptions.

It is important to differentiate between a temporary spike and a step change in intakes. Shelters may informally reason that, when intakes rise (permanently) and the number of adoptions and transfers remains about the same, the census permanently increases to a new steady state. This is incorrect. Rather, in this scenario, either the increase in intakes translates to an equal increase in euthanasia or the census rises monotonically with no upper bound, a practical impossibility. Understanding how temporary spikes in intakes differ from step changes to a new persistent level of intakes helps shelters anticipate these effects and make plans to resolve (reverse) or adapt to such changes.

Our results do not contradict gradual census changes over time. Rather, the results suggest that such changes are much smaller and much slower than a simplistic extrapolation of extra intakes (or reduced outcomes) directly translating to permanent census changes of the same magnitude. As a simplified illustration, assume an LOS of 15 days (which implies a steady-state census equal to half the monthly intakes). Suppose intakes are elevated by 20% for six months, with the census gradually rising by 10%. The census change scaled by monthly flow is 0.05, but the cumulative sum of the excess intakes is 1.2. There is census drift, but most of the additional intakes are absorbed by outcomes.

#### 4.2.2. Stochastic View

Random fluctuations in intakes and adoptions, even without a change in the average rates, cause imbalances. If individual animals arrive independently of each other, intakes in a time period follow a Poisson distribution with mean and variance proportional to the period duration. Similarly, outcomes of interest (adoptions and transfers) occurring independently and at fixed rates also follow a Poisson distribution.

If outcomes are independent of intakes, and the means (Poisson rates) balance out, the cumulative imbalance over any period has a mean of zero. But the variance of the imbalance grows linearly with duration and is unbounded. This either makes the census wildly variable and unpredictable or, similarly to the discussion of dynamics, requires euthanasia and the LRR to become variable and unpredictable over long periods (unless the shelter can intervene and change the underlying random process).

To illustrate, suppose mean daily intake and outcome rates are 1, mean LOS is 13.5, and the census starts at its mean value of 13.5. The annual standard deviation of intakes is 365^½^ = 19.1, and the standard deviation of the annual imbalance is 2^½^ × 19.1 = 27. The census after one year has a mean of 13.5 but a standard deviation of 27. So random fluctuations alone imply implausible variation in the census (which would presumably lead to routine euthanasia for space). Such instabilities are not observed in practice.

#### 4.2.3. Consequences of Dynamic or Stochastic View

The results of this study show that live outcomes change with the census. With this element of flexibility in outcomes, the dynamic and the stochastic view yield behavior that aligns more closely with what we see in shelters. Under this model, a temporary spike in intakes (whether part of a stochastic process or as an external one-time event, e.g., weather) leads to a temporary change in census that eventually resolves, primarily via outcomes. A permanent change in intake (such as an expansion of the geographical jurisdiction of a GRO) leads to a permanent change in the census but not an unbounded growth in the census. In both cases, LRR may be impacted, but nonlive outcomes do not need to absorb a major portion of the change in intakes (let alone the entirety of the change). From the stochastic viewpoint, if the rate at which live outcomes occur depends on the census (while still being stochastic), variations in the census or LRR over longer periods may exist but are dampened. This does not mean that increases in intakes are painless or inconsequential, or that reductions in intakes do not have benefits, but rather that impacts are dampened (via some unknown combination of shelter decisions, unintentional changes in shelter practices, and the behavior of external actors like adopters or rescue partners).

To resolve the impasse in the dynamic and stochastic view outlined above (unbounded census growth or diversion to euthanasia), shelters may attempt to reduce or curtail non-mandated intake categories. The results of our study, however, show such changes playing only a minor role. Notably, changes in the LRR have also been shown to be associated predominantly with outcome rather than intake changes [5]. One-time policy shifts (to add or remove routine relinquishment by owners) that are not connected to census changes do occur. But they are, by definition, sparse and are not likely to be a major regulating factor.

While, for the sake of clarity, we draw a dichotomy between brief spikes in intakes and a permanent change to a new level of monthly intakes, in practice there are also changes that take place over unknown or intermediate durations. The consequences of such shifts would conceptually fall between the cases discussed above. An intermediate-duration rise in intakes would lead to a larger and more durable census increase and would take accordingly longer to resolve (potentially long enough to be, in practice, similar to a permanent change). If the cumulative census change is large, the dynamics would be nonlinear and outside the range of changes we considered here.

### 4.3. Limitations

#### 4.3.1. Data Limitations

As discussed in other studies involving SAC data [5,6], certain limitations flow from the fact that the data are self-reported, and organizations that opt to participate may differ in available resources, level of transparency, data proficiency, and interaction with national organizations, including SAC. Biases by reporting period may also exist, i.e., the beginning, end, or a gap in monthly reporting may be biased. Though SAC fields allow shelters to report young and adult animals separately, many shelters do not. Our study used age-undifferentiated totals and does not capture age effects.

We separate organizations based on their relationship to local governments. It is reasonable to assume that GROs are less susceptible to self-reporting bias [5]. The results for NGO agencies may be less representative. NGOs lose more reports to our data filters and end up with shorter sequences per organization (15.3 vs. 19.6, Table 1).

#### 4.3.2. Scope and Statistical Limitations

Our study is limited to monthly reports above a threshold of intakes and outcomes, i.e., excludes the smallest organizations (one monthly period at a time). Since we exclude large cumulative census changes, our study may not apply to larger, less common changes, which likely have nonlinear effects. For instance, a census shock induced by a large seizure case may have different dynamics. Natural disasters like hurricanes or wildfires may entail high numbers of owner relinquishments and returns. An outbreak of a transmittable disease (in the region or in the shelter) may disrupt intakes (if organizations close to new intakes) and outcomes (if existing animals are quarantined), causing changes and imbalances of a magnitude beyond the scope of this study. The nonlinear effect of larger perturbations may depend on the nature of the precipitating event (e.g., hurricane, disease outbreak, wildfire) and may require different data sets and statistical methods.

Because the cumulative imbalance is constructed from past values of the same intake and outcome counts that serve as dependent variables, its combination with organization-specific intercepts can induce dynamic-panel bias in short panels [21]. This bias, which could potentially overstate the speed at which census changes dissipate, diminishes with sequence length. A mitigating factor is that our average sequence lengths (19.6 for GROs and 15.3 for NGOs) are much longer than the estimated time scales. As a robustness test, we re-examined the cumulative regression for sequences containing a minimum of 12 months. Only small changes were observed, with the cumulative imbalance in GROs dissipated by −0.340 (from −0.325 in our main regression), which shortens the relaxation time constant to 2.4 months (from 2.5). For NGOs, the values likewise indicate a slight acceleration for these longer sequences, with total monthly dissipation −0.452 (from −0.439) and the time constant unchanged (1.7 months). If dynamic-panel bias were material, longer panels would show slower dissipation, so the observed slight acceleration is directional evidence that the bias is not driving the results of this study.

The one-month interval of the reports means that we are not modeling effects that occur at faster time scales, such as a week. While we implicitly extrapolate dissipation to multi-month periods, we did not test such periods directly.

Our model includes a cross-shelter monthly term, which can account for seasonal effects affecting all shelters at the national scale (separately for GROs and NGOs). Other effects may exist that are external to the shelter but not national in scale, and, depending on their form, such effects may bias the results.

#### 4.3.3. Interpretive Limitations

Fundamentally, the study cannot differentiate among proactive shelter decisions, implicit or subconscious policy shifts at the margin (such as interpreting the criteria for adoption or euthanasia more broadly when the census is higher), and the response of external agents such as adopters.

Our study computes averages across many shelters, yielding a national reference. An individual shelter may experience census effects that deviate in magnitude and direction. There is diversity among shelter resources, policies, leadership, facilities, training, partner networks, and community and local government attitudes. The relationship would be difficult to determine in the inherently small sample of a single shelter. Note that we are modeling the whole population, not individual animals. The observational unit is a time interval, not an individual animal, and statistical limitations cannot be overcome by studying a large shelter.

### 4.4. Extensions

Much work remains to be done to understand the movement of animals into and out of shelters. A refined model that allows for seasonal effects can be developed to study cat and kitten censuses.

Models could capture the dynamic effect of an intake or outcome (of a specific type) on the census, intakes, and outcomes as a function of time.

The slopes *K* can be allowed to interact with shelter characteristics, whether exogenous (e.g., community demographics) or endogenous to shelter statistics (e.g., the relative proportions of intake types or additional information on the intake population).

Nonlinear effects that depend on the magnitude of the census change can be incorporated into models such as the one in this study, and such models may distinguish between census increases and decreases. More sophisticated models could also disaggregate past imbalances by their timing and source.

A different methodology could be devised for animal-level data across multiple shelters to study the census, intake, and outcome relationships on time scales shorter than one month. With animal-level data, the census, intakes, and outcomes can be modeled for sub-populations of animals (e.g., slow-track and fast-track animals that a shelter may target [22]), which may be independent or interacting.

## 5. Conclusions

We analyzed monthly shelter statistics using a concurrent model (Equation 2) that statistically decomposes one month’s census change into contributions of each intake and outcome category and a cumulative model (Equation 1) that estimates the effect of past census changes on future intakes and outcomes. The fundamental difference between the two models is, mathematically, the temporal relationship between the flows and the census. In US animal shelters, the two directions of influence between dog census and flows are asymmetric. Within a single month, the decomposition of census changes shows larger components for intakes than for outcomes, with adoption the only outcome type contributing substantially. Census changes accumulated over several months, by contrast, are resolved predominantly through elevated live outcomes and especially adoptions, rather than through reduced intakes, at an implied time constant of 2.5 months for GROs and 1.7 months for NGOs. Because, at least for GROs, this time scale far exceeds typical lengths of stay, per-animal outcome rates evidently depend on census, with average length of stay rising as census increases. For GROs, this is accompanied by a marginal live release rate below the bulk average, while for NGOs, the two rates are essentially equal. Returns to owners have small contributions throughout the analysis, indicating they neither drive census changes nor respond to them. Taken together, these findings argue against treating adoption and transfer capacity as fixed; they support a dynamic and stochastic view of shelter populations in which census perturbations are dampened rather than permanent or unbounded. The study provides a quantitative benchmark that shelters and national organizations can use to understand what drives census changes, how census changes propagate, and how shelters can respond to changes earlier and more proactively.

## Figures and Tables

**Table 1:** Descriptive statistics of intake and outcome types.

	Mean	Std	Min	Median	Max
**GRO (Government-related)**					
stray_at_large	0.587	0.286	0.000	0.596	6.471
relinquished_by_owner	0.235	0.194	0.000	0.200	3.326
remaining_in	0.185	0.242	0.000	0.105	4.149
adoption	0.452	0.250	0.000	0.438	2.537
returned_to_owner	0.274	0.188	0.000	0.245	4.601
noncomm_live_out	0.168	0.229	0.000	0.083	4.560
nonlive_out	0.098	0.114	0.000	0.063	1.431
imbalance	0.015	0.204	−2.050	0.005	3.405
imbalance_cumsum	0.005	0.188	−0.400	0.005	0.400
counts by organization_id	19.6	11.0	2	19	36
counts by period	451.6	70.6	312	477	540
**NGO (Non-government)**					
stray_at_large	0.166	0.261	0.000	0.043	2.594
relinquished_by_owner	0.335	0.295	0.000	0.268	5.144
remaining_in	0.499	0.420	0.000	0.449	7.288
adoption	0.806	0.353	0.000	0.821	8.557
returned_to_owner	0.053	0.118	0.000	0.000	1.724
noncomm_live_out	0.099	0.267	0.000	0.000	5.859
nonlive_out	0.034	0.058	0.000	0.013	1.055
imbalance	0.008	0.316	−2.019	0.000	3.445
imbalance_cumsum	−0.004	0.200	−0.400	−0.005	0.400
counts by organization_id	15.3	10.3	2	12	36
counts by period	358.5	53.8	224	373	424

All fields other than the counts by organization_id or period are expressed as a fraction of the mean of total intakes and total outcomes over the corresponding organization’s contiguous sequence of monthly reports.

**Table 2:** Concurrent regression results.

	Estimate	std.Error	Statistic	*p*-Value	CI	
**GRO (Government-related)**						
stray_at_large	0.222	0.013	17.278	<0.001	0.197	0.247
relinquished_by_owner	0.139	0.012	11.833	<0.001	0.116	0.162
remaining_in	0.247	0.024	10.143	<0.001	0.199	0.295
adoption	−0.216	0.011	−18.970	<0.001	−0.239	−0.194
returned_to_owner	−0.028	0.008	−3.488	<0.001	−0.044	−0.012
noncomm_live_out	−0.121	0.012	−9.982	<0.001	−0.145	−0.097
nonlive_out	−0.027	0.005	−5.447	<0.001	−0.037	−0.017
**NGO (Non-government)**						
stray_at_large	0.095	0.009	10.645	<0.001	0.078	0.113
relinquished_by_owner	0.194	0.012	16.424	<0.001	0.170	0.217
remaining_in	0.452	0.017	27.309	<0.001	0.419	0.484
adoption	−0.197	0.011	−18.105	<0.001	−0.219	−0.176
returned_to_owner	−0.004	0.002	−2.209	0.027	−0.007	−0.000
noncomm_live_out	−0.054	0.008	−6.497	<0.001	−0.070	−0.038
nonlive_out	−0.004	0.002	−2.194	0.029	−0.008	−0.000

Each field was regressed against the same month’s imbalance in a model that includes covariates for each shelter and each month (model 2), but only the slope *K* is reported. The regressions decompose the imbalance into the contributions of intakes and outcomes. Data were equal-weighted, and standard errors used CR1 clustering by organization.

**Table 3:** Cumulative regression results.

	Estimate	std.Error	Statistic	*p*-Value	CI	
**GRO (Government-related)**						
stray_at_large	−0.011	0.013	−0.851	0.395	−0.036	0.014
relinquished_by_owner	−0.017	0.009	−1.845	0.065	−0.036	0.001
remaining_in	−0.013	0.011	−1.120	0.263	−0.035	0.010
adoption	0.156	0.012	13.217	<0.001	0.133	0.179
returned_to_owner	0.014	0.007	1.914	0.056	−0.000	0.028
noncomm_live_out	0.074	0.011	6.728	<0.001	0.052	0.096
nonlive_out	0.040	0.005	7.339	<0.001	0.029	0.051
imbalance	−0.325	0.015	−21.617	<0.001	−0.354	−0.295
**NGO (Non-government)**						
stray_at_large	−0.002	0.008	−0.260	0.795	−0.019	0.014
relinquished_by_owner	−0.033	0.012	−2.818	0.005	−0.056	−0.010
remaining_in	−0.075	0.020	−3.847	<0.001	−0.114	−0.037
adoption	0.264	0.018	14.999	<0.001	0.229	0.298
returned_to_owner	0.008	0.003	2.684	0.007	0.002	0.013
noncomm_live_out	0.046	0.010	4.446	<0.001	0.026	0.067
nonlive_out	0.011	0.002	4.681	<0.001	0.006	0.015
imbalance	−0.439	0.020	−22.284	<0.001	−0.478	−0.400

Each field was regressed against past census changes in a model that allows covariates for each shelter and each month (model 1), but only the slope *K* is reported. The census change was computed from the beginning of the sequence (of a given shelter) to the beginning of the month being regressed. Data were equal-weighted, and standard errors used CR1 clustering by organization.

**Table 4:** Ratio of slope observed in the cumulative regression over a default reference.

	Relative Slope
**GRO (Government-related)**	
stray_at_large	−0.11
relinquished_by_owner	−0.46
remaining_in	−0.42
adoption	2.12
returned_to_owner	0.31
noncomm_live_out	2.72
nonlive_out	2.53
**NGO (Non-government)**	
stray_at_large	−0.06
relinquished_by_owner	−0.45
remaining_in	−0.69
adoption	1.49
returned_to_owner	0.67
noncomm_live_out	2.13
nonlive_out	1.41

The default reference is computed by distributing the observed total impact of cumulative past census change equally between intakes and outcomes and then multiplying by the fractions in the descriptive statistics from Table 1. The sign of the ratio is preserved from its numerator (the observed slope).

## Data Availability

The data were obtained from Shelter Animals Count (now a program of the American Society for the Prevention of Cruelty to Animals). Please inquire with SAC/ASPCA about the conditions under which they make the data available for research.
